# Quantitative analysis of naphthalene, 1-naphthol and 2-naphthol at nanomol levels in geothermal fluids using SPE with HPLC

**DOI:** 10.1016/j.mex.2023.102244

**Published:** 2023-06-12

**Authors:** Lucjan Sajkowski, Terry M. Seward, Bruce W. Mountain

**Affiliations:** a Department of Earth Resources and Materials, GNS Science, Wairakei, New Zealand; b School of Geography, Environment and Earth Sciences, Victoria University of Wellington, New Zealand

**Keywords:** Naphthalene, Naphthol, Chromatography, HPLC, SPE geothermal tracers, SPE-HPLC

## Abstract

Naphthalene (NAP), 1-naphthol (1-NAP) and 2-naphthol (2-NAP) are the thermal decomposition products of naphthalene sulfonates making them potentially new geothermal reservoir permeability tracers, however, to date, no sensitive and fast detection method for these compounds has been developed. In order to facilitate sensitive and rapid analysis of these compounds in geothermal brines and associated steam condensates, a high-performance liquid chromatography (HPLC) method combined with solid-phase extraction (SPE) has been developed.

• A method for determination of naphthalene, 1- and 2-naphthol from brines has been developed.
• These compounds have been detected in steam samples from three New Zealand geothermal fields.
• As breakdown products of NDS/NSA, these compounds have potential use as geothermal tracers.

## Graphical abstract

**Figure:**
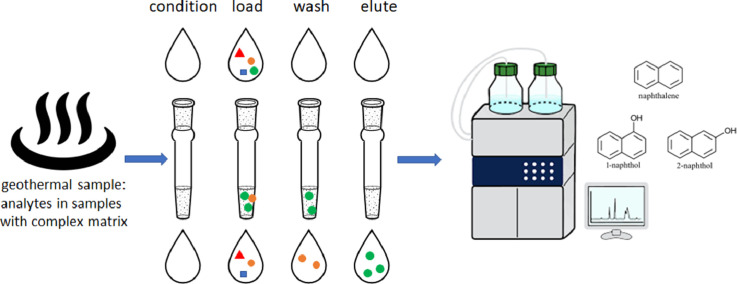

Specifications table

**Table:** 

Subject area:	Earth and Planetary Sciences
More specific subject area:	Geothermal Geochemistry
Name of your method:	SPE-HPLC
Name and reference of original method:	M. Nottebohm, T. Licha, Detection of Naphthalene Sulfonates from Highly Saline Brines with High-Performance Liquid Chromatography in Conjunction with Fluorescence Detection and Solid-Phase Extraction, Journal of Chromatographic Science, Volume 50, Issue 6, July 2012, Pages 477– 481, 10.1093/chromsci/bms029
Resource availability:	Synergi™ 4 µm Hydro-RP 80 Å, LC Column 150 × 4.6 mm https://www.phenomenex.com/products/part/00f-4375-e0?fsr=1 rityGuard™ cartridges for HPLC AQ C18 columns with 3.2 to 8.0mm internal diameters https://www.phenomenex.com.hk/Products/Part/AJ0-7511 Naphthalene solution certified reference material, 200 μg/mL in methanol https://www.sigmaaldrich.com/NZ/en/product/supelco/crm48641 Naphthalene certified reference material, TraceCERT® https://www.sigmaaldrich.com/NZ/en/product/sial/91489 1-Naphthol - ReagentPlus ® , ≥99% - Sigma-Aldrich https://www.sigmaaldrich.com/NZ/en/product/sial/n1000 2-Naphthol - ReagentPlus ® , ≥99% - Sigma-Aldrich https://www.sigmaaldrich.com/NZ/en/product/aldrich/185507 Acetonitrile, HPLC Gradient Grade, Thermo Scientific Chemicals https://www.fishersci.com/shop/products/acetonitrile-hplc-gradientgrade-thermo-scientific/AC325730010 Discovery® DSC-18 SPE Tube 100mg https://www.sigmaaldrich.com/NZ/en/product/supelco/52602u Discovery® DSC-18 SPE Tube 500mg https://www.sigmaaldrich.com/NZ/en/product/supelco/52604u Strata™-X 33 µm Polymeric Reversed Phase, 30 mg / 1 mL, Tubes , 100/Pk https://www.phenpreview.com/Products/Part/8B-S100-TAK Strata™-X 33 µm Polymeric Reversed Phase, 500 mg / 3 mL, Tubes , 50/Pk https://www.phenpreview.com/Products/Part/8B-S100-HBJ

## Method details

 

## Introduction

**Fig. 1:**
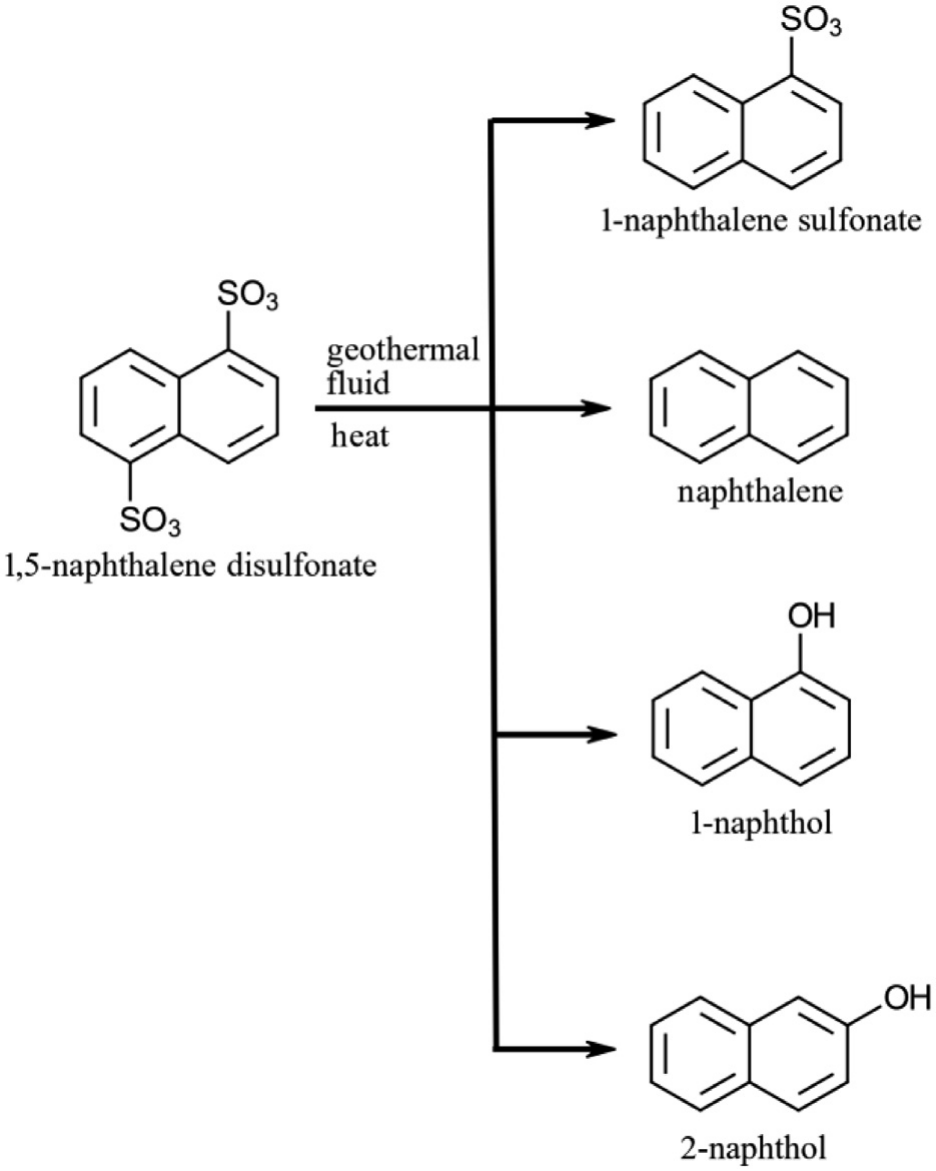
Structure of possible products of 1,5-naphthalene disulfonate decomposition under geothermal conditions and representation of reaction pathways [13].

Naphthalene (NAP), 1-naphthol (1-NAP) and 2-naphthol (2-NAP) are bicyclic hydrocarbons (Fig. 1) used to produce dyes, synthetic rubbers, pesticides, and in the pharmaceutical industry [[1], [2], [3], [4]]. As naphthalene and its derivatives are toxic at elevated levels [5,6], the wide use of these compounds (e.g. tars, asphalt and the like as) means they can be present in the environment as contaminants [[7], [8], [9], [10]]. Naphthalene has also been shown to occur naturally in volcanic gases [11]. Additionally, it has also been proven that NAP, 1-NAP and 2-NAP are formed through thermal degradation of 1,5-naphthalene sulfonates, commonly used tracers by geothermal industry [[12], [13], [14], [15], [16]]. Although, methods for determination of NAP in pure water exist (e.g. [17]), they are not applicable to saline fluids. In order to measure the concentration of these breakdown products in geothermal brines, a reliable detection method (i.e. low detection limits, linearity over a wide range, robustness against salts and pH) is required, with potential useful applications to the geothermal industry.

NAP, 1-NAP and 2-NAP have low solubility in water at ambient temperature (Table 1), however, their solubility increases with temperature increase. Experiments to examine their solubility at elevated temperatures have been conducted. These experiments show an increase in solubility of these compounds as temperature increases up to 75 °C. For example, 2-NAP solubility increases from 4.95×10^−3^ to 4.16×10^−2^ mol kg^−1^ as the temperature increases from 25 to 75 °C [[18], [19], [20]]. The increase in solubility makes these aromatic compounds very mobile within geothermal systems. Naphthalene is a volatile organic compound [21], which will fractionate into the steam phase, therefore may also become a useful indicator of steam formation (i.e. boiling) in active geothermal systems.

**Table 1:** Solubility of NAP, 1-NAP and 2-NAP in water at 25°C.

compound	solubility at 25°C (mol kg^−1^)	source
naphthalene (NAP)	2.36×10^−4^	[22]
1-naphthol (1-NAP)	6.01×10^−3^	[23]
2-naphthol (2-NAP)	4.95×10^−3^	[20]

Current analytical methods used to quantify these compounds are not suitable for rapid and sensitive analysis of geothermal brines. Furthermore, none of the currently established methods target NAP, 1-NAP and 2-NAP in saline solutions simultaneously. Among available methods, the most popular are gas chromatography (GC) and liquid chromatography (LC) with UV and fluorescence detection [[24], [25], [26]]. The GC method with mass spectrometry (MS) has been employed in analyses of environmental river water samples for the presence of NAP [27], but this method does not include 1-NAP or 2-NAP. Other studies describe the LC-UV method that targets 1-NAP and 2-NAP in water samples [4]. An alternative method, cloud point extraction (CPE), prior to performing capillary zone electrophoresis (CZE; to analyse river water samples for naphthol concentrations) was developed by Zhong et al., [28], however, it does not target naphthalene and this method is not sufficiently sensitive for use at geothermal tracer concentrations. For comparison, detection limit of naphthalene in our method is a s low as 0.127 µg kg^−1^ (1.01 nmol kg^−1^), while recently published paper on detection on naphthalene in sea water reported detection limits of 0.76 µg kg^−1^ [29].

The method presented in this chapter involves sample concentration and purification through solid phase extraction (SPE). SPE methods are commonly used for waste waters and river samples [[4], [8]], but these have not been applied to geothermal fluid or steam samples. As there was no available HPLC methods designed to detect sufficiently low concentrations (i.e. at nmol levels) of NAP, 1-NAP and 2-NAP from geothermal fluids, a HPLC with fluorescence detector methodology combined with SPE is proposed.

## Experimental

### Reagents

Naphthalene (>99%,), 2-naphthol (>99%, β-naphthol) and 1-naphthol (>99%, α-naphthol) were purchased from Sigma-Aldrich, acetonitrile (HPLC grade) was obtained from Fischer Scientific GmbH. Ethanol and methanol were HPLC grade from Bio-Strategy. Ultrapure water with a conductivity of 0.055 μS cm^−1^ (18.2 MΩ cm^−1^) was produced by a Arium® pro-ultrapure water system.

### Chromatographic separation

The naphthalene and naphthols are analyzed by high performance liquid chromatography (HPLC) with fluorescence detection using a Shimadzu Prominence RF-20Axs. The separation of the compounds is made using reversed-phase Synergi Hydro-RP column with particle size of 4 µm and pore size of 80 Å, 150×4.6 mm (Phenomenex Inc.) thermostated at 25 °C. The column is protected with a SecurityGuard AQ C18 guard column, 3 µm, 4.0×3.0 mm (Phenomenex Inc.). A 50% (v/v) aqueous acetonitrile solution was used as a carrier phase. The flow-rate is 1.5 ml min^−1^ and 2 ml min^−1^. A 25 µl volume of sample is injected each time and a 25 µl ultrapure water blank is run between each standard/sample. A multiple excitation and emission configuration is used. The detector operates at excitation wavelengths of 219 nm and 254 nm and emission wavelengths of 330 nm and 445 nm as shown in Table 2. The sample was injected by autosampler.

**Table 2:** The HPLC analysis configuration.

Time (min)	Flow (ml min^−1^)	Excitation (nm)	Emission (nm)
0 - 5	1.5	219	445
5 - 13	2.0	254	330

### Standard preparation and calibration procedure

The stock solution for calibration standards is prepared by spiking ethanol with a mixture of naphthalene (7.80×10^−5^ mol kg^−1^), 1-naphthol and 2-naphthol (6.94×10^−5^ mol kg^−1^). Eight calibration solutions are selected in the range 2.9–1170 nmol kg^-1^ (Table 3). The calibration solution 1 - 5 were prepared stepwise. To prepare the calibration 1 – 3 solution 5 was used, while 7 was diluted to prepare solutions 4 and 5. Unspiked HPLC grade ethanol is used as a blank. Linear calibration curves are obtained by plotting the quotients of the integrated peaks as a function of the standard concentrations. These graphs were used to determine the unknown naphthalene and naphthol concentration in the brine samples. The calibration curves give good linearity with correlation coefficients (R^2^) between 0.9992 and 0.9998.

**Table 3:** Calibration solutions concentration.

Standard	Concentration (nmol kg^-1^)
1	2.90
2	15.4
3	76.4
4	120
5	245
6	548
7	840
8	1170

### Solid-phase extraction

To isolate, enrich and increase analyte recovery, a solid phase extraction method was developed. Three different SPE cartridges were tested. Two of them with the same polymer base but different amounts of sorbent, (i.e. Discovery DSC-18 with either 100 mg and 500 mg sorbent from Sigma-Aldrich and Strata-X 33 µm Polymeric Reversed Phase, 30 mg from Phenomenex. Before using the cartridges, they were eluted with ethanol and then conditioned with water (Table 4) after which, 1 ml of sample (i.e. spiked sample) was loaded. The cartridges are dried with nitrogen gas for 5 min (20 psi and 2.0 l min^−1^). Subsequently, the analyte was eluted with ethanol (or methanol) and collected into a vial and analyzed with HPLC.

**Table 4:** Parameters used during evaluation of SPE protocol. The suggested optimum version has been highlighted.




*as the wash solution: 30:70, v/v, ethanol/water solution was used, or: 40:60, v/v, methanol/water.

**eluent used in this study: 99.9% ethanol or 100% methanol HPLC grade.

To improve SPE performance, all steps (conditioning, sample load, wash, elute, modifiers) had to be adjusted. As these steps can influence extraction efficiency, all were investigated for each kind of sorbent. The method was tested using a sample of geothermal brine (pH ∼ 5.5) spiked with a calculated concentration of NAP, 1-NAP and 2-NAP (each 10 ug kg^−1^) and in laboratory prepared solutions with neutral pH and different salt concentrations of 0.00, 0.05, 0.50 and 1.00 mol kg^−1^ NaCl.

**Fig. 2:**
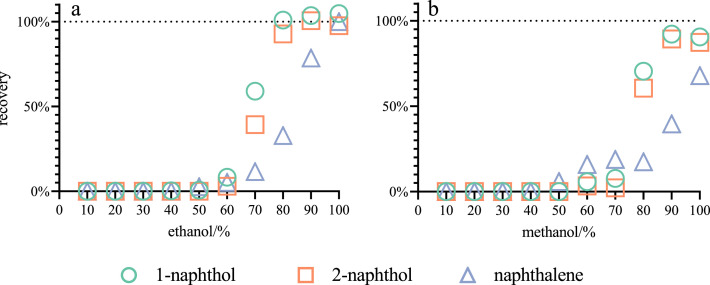
Effect of aqueous dilution of methanol and ethanol on the analyte recovery using SPE (a) ethanol and (b) methanol on the analyte extraction. SPE cartridges: 100 mg Discovery-C18.

One of the goals was to obtain the highest recovery from SPE. The elution is critical for successful SPE process and choosing the right organic solvent is important for the elution preference. Two available solvents were chosen (i.e. methanol and ethanol) and tested at different concentrations (from 10% to 100%; Fig. 2). Results show a recovery increase when ethanol is used in comparison to methanol, thus ethanol was chosen as the eluent. In addition, 30% ethanol solution was used as the wash solution, as it was the highest concentration of the organic solvent which did not elute targeted compounds. To try to minimise any contamination from plastic (i.e. pipettes, storage containers etc.) samples and standards are stored in glass bottles/vials and solutions handled with glass pipettes.

Among different SPE cartridges, the Discovery-C18 (100 mg) cartridge give the most effective (almost complete) recovery (Table 5). Satisfactory results are also observed on the Strata-X 33 µm (30 mg) cartridge. The cartridge Discovery-C18 (500 mg) provided low recoveries, while Strata-X 33 µm (500 mg) retains all analyzed compounds (i.e. no recovery). The Discovery-C18 (100 mg) was selected as the most effective (i.e. high recoveries) and further employed as the SPE cartridge for this method.

**Table 5:** Recovery and relative standard deviation (RSD) of naphthalene, 1-naphthol and 2-naphthol in percentages for different solid phase cartridges considered in this study (10 ug kg^−1^ of each compound added to 1 ml samples).

	Discovery DSC-18, (100 mg)		Discovery DSC-18, (500 mg)	
compound	recovery [%]	± RSD [%]	recovery [%]	± RSD [%]
1-naphthol	99.58	0.30	94.15	12.10
2-naphthol	99.73	0.19	84.24	4.26
naphthalene	99.92	0.05	96.77	2.32

	Strata-X 33 µm, (30 mg)		Strata-X 33 µm, (500 mg)	
compound	recovery [%]	± RSD [%]	recovery [%]	± RSD [%]

1-naphthol	98.52	1.06	0.00	-
2-naphthol	99.52	0.49	0.00	-
naphthalene	96.00	2.88	0.36	-

### The effect of salinity on HPLC results

The total salinity of a geothermal fluids differs for different geothermal fields. It can be low (e.g. 0.04 mol kg^−1^ for Rotokawa, New Zealand; [30], 0.23 mol kg^−1^ in Bacman Geothermal Field, Philippines; [31]) or as high as 1 mol kg^−1^ in Reykjanes, Iceland [32] or Salton Sea, USA, where NaCl = 4.8 mol kg^−1^ [33]. It was previously reported that high salt concentration has a negative influence on the analysis of naphthalene sulfonates [34] and other analytes [4]. The HPLC method presented in this chapter is designed to analyse low concentrations of NAP, 1-NAP and 2-NAP (Table 5) in saline solutions (≤ 1.00 mol kg^−1^).

**Fig. 3:**
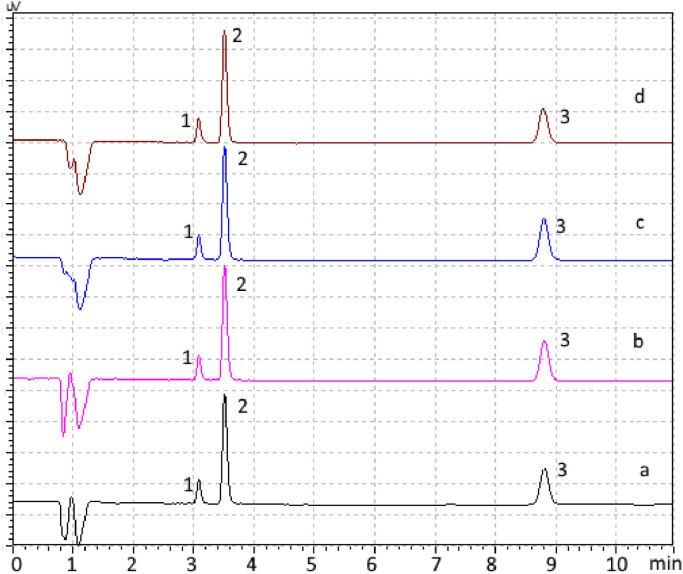
Chromatogram of a 10 ug kg^−1^ mix-standard consisting of 2-naphthol (peak 1), 1-naphthol (peak 2) and naphthalene (peak 3) in NaCl = 1.00 mol kg^−1^(a), NaCl = 0.50 mol kg^−1^ (b), NaCl = 0.05 mol kg^−1^ (c) and distilled water (a) using a Phenomenex Synergi Hydro-RP C18 column and direct injection.

Three different background salt concentrations (i.e. 0.05 mol kg^−1^, 0.50 mol kg^−1^ and 1.00 mol kg^−1^ NaCl) and distilled water are employed. The experimental data shows that there is no loss in resolution with the different salt matrices during analysis (Fig. 3) and no further need for other sample treatment is required.

### Quality assurance and data treatment control

To ensure high quality data diluted in ethanol NAP certified reference material, TraceCERT® and 1-NAP and 2-NAP ReagentPlus®, ≥99% supplied by Sigma Aldrich were analyzed during each HPLC-SPE run.

Method validation was performed according to the guidelines set by the United States Environmental Protection Agency. Method detection limit (MDL) refers to the smallest concentration of a substance that can be measured by an analytical procedure with 99% confidence that the analyte concentration is greater than zero [[35], [36]]. The MDL is calculated using the formula:

(1) $$\text{MDL}=s\cdot t_{\;(n\;-\;1,\;1\;-\;\alpha =0.99)}$$

where, n is number of replicate spike determinations, s is standard deviation of measured concentrations of n spike determinations, t is the Student's value at n - 1 degrees of freedom and 1- α refers to the 99% confidence level.

Minimum level (ML) is the lowest concentration of an analyte which can be determined with the method and constitutes a calibration point [[35], [36]]. The ML is defined as,

(2) ML=3.18·MDL

Repeatability and recovery were validated through several runs by replicate (n = 15) analyses of spiked ethanol at different concentration levels (Table 5). Calibration curves for NAP, 1-NAP and 2-NAP are linear with correlation coefficients > 0.999. Method Detection Limit and Minimum Level are presented in Table 6. Whereas, the details for calculations of MDL and ML are given in Appendix 1.

**Table 6:** Calibration Parameters of the overall method, including SPE and HPLC detection.

compound	linear range nmol kg^−1^	MDL nmol kg^−1^	ML nmol kg^−1^
1-naphthol	4.86 - 1.04	1.11	3.61
2-naphthol	4.86 - 1.04	1.04	3.40
naphthalene	6.24 - 1.17	1.01	3.12

MDL = method detection limit; ML = minimum level.

## Conclusions

This study describes a sensitive method for determination of naphthalene, 1-naphthol and 2-naphthol from saline brines (up to 1.00 mol kg^−1^ NaCl) using HPLC-fluorescence detector combined with SPE methodology. Among different tested SPE cartridges, the Discovery-C18 (100 mg) cartridge was the most effective. Ethanol has been selected as the best eluent. While there was no effect of salinity in range 0.00 - 1.00 mol kg^−1^ NaCl on quality of analysis. However, future tests are required to examine effect of geothermal fluid pH on SPE process.

The method can be applied in future hydrogeological and permeability tracer tests in geothermal reservoirs. Measurable amounts of naphthalene and 1- and 2-napthol have been successfully detected in geothermal steam samples from different New Zealand geothermal fields using the analytical the method presented in this study (Table 7).

**Table 7:** Concentration (mol kg^−1^) of NAP, 1-NAP and 2-NAP measured in steam condensate samples collected from different active geothermal fields.

compound	Site 1	Site 2	Site 3
1-naphthol	1.25×10^−6^	9.02×10^−7^	1.94×10^−6^
2-naphthol	2.57×10^−6^	n.d.	n.d.
naphthalene	3.67×10^−5^	8.50×10^−5^	5.09×10^−4^

n.d. = not detected.

## CRediT authorship contribution statement

**Lucjan Sajkowski:** Conceptualization, Methodology, Data curation, Writing – original draft. **Terry M. Seward:** Writing – review & editing, Supervision. **Bruce W. Mountain:** Writing – review & editing, Supervision.

## Declaration of Competing Interest

The authors declare that they have no known competing financial interests or personal relationships that could have appeared to influence the work reported in this paper.

## Data Availability

Some data like filed locations are confidential.

## Appendix B Supplementary materials

Image, application 1
