# Supplementary material for: Quantitative analysis of naphthalene, 1-naphthol and 2-naphthol at nanomol levels in geothermal fluids using SPE with HPLC
Source: MethodsX. 2023 Jun 12;11:102244. doi: 10.1016/j.mex.2023.102244 (PMC10300394; doi:10.1016/j.mex.2023.102244)
Supplement: Supplementary file 1 [file mmc1.docx]

**Supplementary material *and/or* additional information [OPTIONAL]**

Appendix 1 Method Detection Limit Calculations

| **MDL Calculation** | |  |  |  |
| --- | --- | --- | --- | --- |
| Analyte | | 2-naphthol | 1-naphthol | naphthalene |
| Spike Concentration | | 0.7 | 0.7 | 0.8 |
| Concentration Units | | µg kg^-1^ | µg kg^-1^ | µg kg^-1^ |
| Analysis Date | Replicate Number |  |  |  |
| 11/04/2019 | 1 | *0.6353* | *0.7008* | *0.8358* |
| 11/04/2019 | 2 | *0.7248* | *0.6824* | *0.8183* |
| 12/05/2019 | 3 | *0.7825* | *0.7537* | *0.8073* |
| 12/05/2019 | 4 | *0.8650* | *0.8877* | *0.9594* |
| 12/05/2019 | 5 | *0.8200* | *0.7369* | *0.8808* |
| 12/05/2019 | 6 | *0.7663* | *0.8288* | *0.8795* |
| 12/05/2019 | 7 | *0.7286* | *0.8177* | *0.8259* |
| 15/05/2019 | 8 | *0.8084* | *0.8042* | *0.8188* |
| 15/05/2019 | 9 | *0.7724* | *0.8117* | *0.8796* |
| 15/05/2019 | 10 | *0.7077* | *0.7324* | *0.8293* |
| 15/05/2019 | 11 | *0.8278* | *0.8364* | *0.806* |
| 15/05/2019 | 12 | *0.7279* | *0.7272* | *0.9227* |
| 08/07/2019 | 13 | *0.7379* | *0.8218* | *0.8936* |
| 08/07/2019 | 14 | *0.7391* | *0.8766* | *0.7885* |
| 08/07/2019 | 15 | *0.705* | *0.7854* | *0.8411* |
| Mean (Average) | | 0.75658 | 0.78691 | 0.85244 |
| Standard Deviation | | 0.05830 | 0.06209 | 0.04823 |
| **Method Detection Limit (MDL)** | | **0.153** | **0.163** | **0.127** |
| Limit of Quantification (LOQ) | | 0.58297 | 0.62086 | 0.48233 |
| Uncertainty of Measurement (UoM) | | 15.4% | 15.8% | 11.3% |
| High Spike Check | | OK | OK | OK |
| Low Spike Check | | OK | OK | OK |
| S/N (2.5 to 10) | | 13.0 | 12.7 | 17.7 |
| Replicate Recovery (85% to 115%) | | 108% | 112% | 107% |
